# (Cryo)Transmission Electron Microscopy of Phospholipid Model Membranes Interacting with Amphiphilic and Polyphilic Molecules

**DOI:** 10.3390/polym9100521

**Published:** 2017-10-19

**Authors:** Annette Meister, Alfred Blume

**Affiliations:** 1 Institute of Chemistry, Martin Luther University Halle-Wittenberg, D-06120 Halle (Saale), Germany; alfred.blume@chemie.uni-halle.de; 2 Institute of Biochemistry and Biotechnology, Martin Luther University Halle-Wittenberg, D-06120 Halle (Saale), Germany

**Keywords:** lipids, fluorinated amphiphiles, bolalipids, polyphiles, (cryo)transmission electron microscopy

## Abstract

Lipid membranes can incorporate amphiphilic or polyphilic molecules leading to specific functionalities and to adaptable properties of the lipid bilayer host. The insertion of guest molecules into membranes frequently induces changes in the shape of the lipid matrix that can be visualized by transmission electron microscopy (TEM) techniques. Here, we review the use of stained and vitrified specimens in (cryo)TEM to characterize the morphology of amphiphilic and polyphilic molecules upon insertion into phospholipid model membranes. Special emphasis is placed on the impact of novel synthetic amphiphilic and polyphilic bolalipids and polymers on membrane integrity and shape stability.

## 1. Introduction

Biological membranes are composed of a complex mixture of lipids, proteins, and carbohydrates forming a permeability barrier for cells and cell organelles. The lipids usually organize in bilayer geometry with asymmetric composition, thereby providing the matrix for embedded integral or peripheral membrane proteins [1,2,3]. Nowadays, transmission electron microscopy (TEM) techniques resolve structural details of membrane proteins and protein complexes that are too large for nuclear magnetic resonance (NMR) spectroscopy, or are too flexible for X-ray analysis and cannot form crystals [4,5]. The “resolution revolution” was mainly due to advances in microscope instrumentation, imaging technology, and computation, enabling the generation of protein structures with almost atomic resolution [6,7,8,9,10,11,12]. High-resolution TEM applications such as electron cryo tomography [13], single-particle analysis [14], and 2D electron crystallography [15], together with the focused-ion-beam milling technique for specimen preparation [16,17] and correlated fluorescence and 3D electron microscopy [18,19], considerably improved our understanding of the composition and function of biological membranes.

Because of the complexity of biological membranes, artificial model membranes are frequently applied for simplification. Being composed of saturated or unsaturated phospholipids, they facilitate investigations on the impact of surfactants, peptides, proteins, polymers, drugs, or other guest molecules on membrane integrity. CryoTEM is well suited to study membrane integrity as well as shape changes due to the attachment or incorporation of natural or synthetic guest molecules. Vesicles are frequently used artificial model systems that can be visualized before and after incubation with guest molecules. Here, cryoTEM exhibits special key benefits compared with X-ray and neutron diffraction or scattering, as well as NMR spectroscopy. TEM provides images of heterogeneous aqueous suspensions that contain aggregates in the nanometer range with various sizes and shapes. The aggregates might be composed of lipids, surfactants, liquid crystals, peptides, proteins, polymers, drugs, and mixtures which form aggregate structures such as vesicles, fibers, discs, or micelles. Even mixtures of these aggregates can easily be distinguished [20,21,22,23,24,25,26].

In this review, we report on the use of stained and vitrified specimens for (cryo)TEM ((cryo)TEM stands for cryoTEM with vitrified samples or TEM using stained samples) to visualize phospholipid model membranes upon interaction with amphiphilic and polyphilic guest molecules. Whereas amphiphiles easily insert into lipid bilayers because of their hydrophilic and lipophilic parts, polyphiles contain additional mesogenic (i.e., liquid-crystal-forming) or fluorinated parts that might cause self-assembly within the membrane. As a consequence, either membrane stabilization or changes in membrane shape are expected which can be visualized by (cryo)TEM. We will not cover freeze–fracture electron microscopy (FFEM) but will occasionally refer to results from this technique. All of the TEM images shown are obtained from samples resulting from collaborations within the Research Unit Forschergruppe FOR 1145 funded by the German Research Foundation (Deutsche Forschungsgemeinschaft, DFG) and the French–German FLUOR initiative funded by the Agence Nationale de la Recherche (ANR) and the DFG.

## 2. TEM Preparation Techniques

### 2.1. Preparation of Stained Samples for TEM

Staining of self-assembled aggregates in aqueous suspension is an easy and rapid method, where the specimen is incubated with the staining solution (1 to 3% (*w*/*v*)) of uranyl acetate (UA), uranyl formate (UF), ammonium molybdate (AM), sodium phosphotungstate (SPT), or phosphotungstic acid (PTA). These staining agents are either dissolved in water [27,28] or organic solvents such as tetrahydrofuran (THF), dimethyl formamide (DMF), or dimethyl sulfoxide (DMSO) [29]. The pH range useful for aqueous staining agents varies between pH 3–4 for UA and UF, pH 5–8 for AM, and pH 4–9 for SPT and PTA [30]. PTA has a significant disruptive effect on many membrane systems. Similar to AM and SPT, it produces less contrast than UA and UF. UA it is not recommended for use with specimens that are unstable in acidic conditions. Furthermore, this stain precipitates at physiological pH and in the presence of high salt concentrations. Then, additional washing steps with water have to be applied before addition of the staining agent. However, UA gives images with finer grain than those using other staining agents, except UF [31], and can be stored in the dark at 4 °C for more than a year [32].

**Figure 1:**
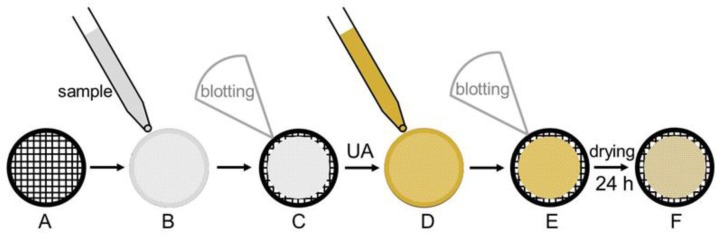
Schematic representation of the staining procedure according to Harris [27]. (**A**) Formvar coated Cu-grid. (**B**) Sample application. (**C**) Blotting. (**D**) Application of uranyl acetate (UA). (**E**) Blotting. (**F**) Drying.

A typical staining procedure is depicted in Figure 1. A Cu grid (A), coated either with a Formvar polymer film or a continuous carbon film is used as sample support. The carbon film has to be treated under partial vacuum with a plasma induced by a glow-discharge to create a hydrophilic carbon surface [27]. Stained specimens are prepared by spreading 5 µL of the sample suspension onto the grid (B). After 1 min, excess liquid is blotted off with filter paper (C), and 5 µL of a 1% (*w*/*v*) aqueous uranyl acetate solution is placed onto the grid (D) and drained off after 1 min (E). Finally, the specimen is dried for 24 h at least (F) before examination with the electron microscope.

**Figure 2:**
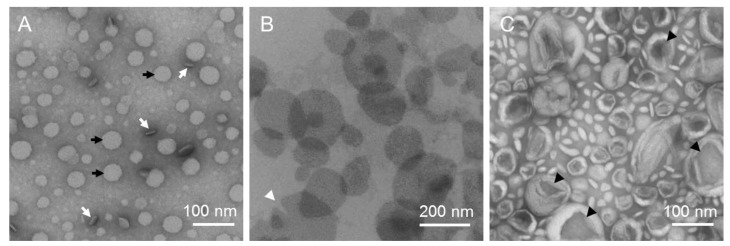
(**A**) Negatively stained bolalipid/1,2-dipalmitoyl-*sn*-glycero-3-phosphocholine (DPPC) discs [35]. (**B**) Positively stained bolalipid/1,2-dimyristoyl-*sn*-glycero-3-phosphocholine (DMPC) discs [36]. (**C**) DMPC vesicles that collapsed because of drying.

Ideally, the stain should not react with or bind to the specimen. If there is no reaction, then the background of the image is dark and the aggregates appear brighter. Figure 2A shows this classic case of “negative staining” [33]. However, uranyl cations will bind to negatively charged proteins, lipids, and nucleic acid phosphate groups [34]. In this case, the aggregates are darker than the background and the procedure is called “positive staining” (Figure 2B). Sometimes, protein dissociation, protein flattening, or uneven negative stain distribution across the protein sample occurs that prevents the processing of the images for a meaningful three-dimensional image reconstruction.

The staining procedure has the drawback that aggregates might be uniformly oriented or crushed during drying of the specimens. Discs will preferentially orient in face-on (black arrows) or edge-on (white arrows; Figure 2A) orientation. Lipid vesicles usually do not stay spherical but collapse because of the evaporation of water (black arrowheads; Figure 2C). If the vesicle bilayer is smooth, that is, after incorporation of detergent, then the drying vesicle flattens to a pancake-like shape that is difficult to distinguish from a disc. However, the appearance of “pacman”-like shapes (white arrowhead; Figure 2B) is a strong hint to vesicles breaking up during drying.

Another possibility to decide whether discs or vesicles are present is to prepare vitrified samples for cryoTEM, where the shape of the aggregates is preserved in a layer of amorphous ice. However, if access to an electron microscope equipped with a cryo sample holder is limited, preliminary tests with stained samples might be helpful.

### 2.2. Preparation of Vitrified Samples for CryoTEM

**Figure 3:**
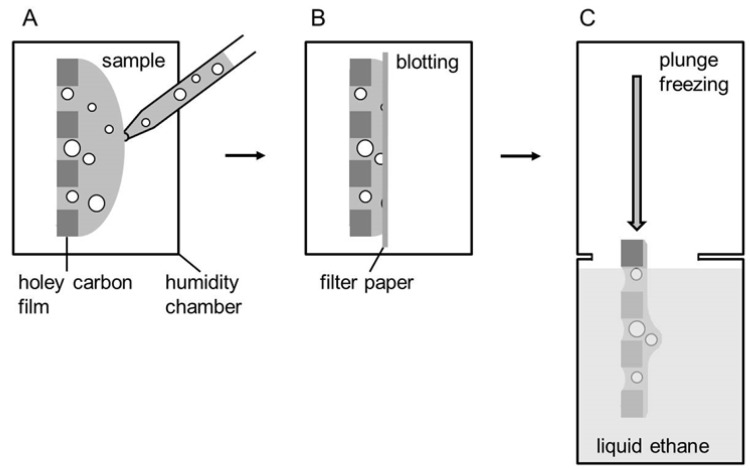
Schematic representation of the plunge freezing procedure according to Resch et al. [41]. (**A**) Sample application to the grid in a humidity chamber. (**B**) Blotting. (**C**) Plunge freezing into liquid ethane.

Vitrified specimens for cryoTEM are usually prepared by a blotting procedure performed in a chamber with controlled temperature and humidity (Figure 3) [37,38,39]. A temperature- and humidity-controlled environment is essential to prevent osmotic and temperature-induced alterations involving a thermotropic lipid phase change [40]. About 3 µL of the aqueous sample solution (1 mg∙mL^−1^) are placed onto a glow-discharged electron microscopy (EM) grid coated with a holey carbon film (A). Excess solution is then removed with a filter paper, leaving a thin film of the solution spanning the holes of the carbon film on the EM grid (B). Vitrification of the thin film is achieved by rapid plunging of the grid into liquid ethane held just above its freezing point (C) [41,42]. The vitrified specimen is kept below 108 K during storage, transfer to the microscope, and investigation.

By applying this procedure, the sample suspension is vitrified and the shape of the aggregates is preserved in an up to 500 nm thin layer of amorphous ice within the holes of the carbon film [43]. Larger aggregates tend to cluster at the edges of the holes, where the thickness of the amorphous ice layer is higher. Since both the embedded aggregates and the vitrified matrix are sensitive to electron-beam-induced changes, low-dose techniques should be applied to reduce damage by electron irradiation [44,45].

Besides NMR spectroscopy, cryoTEM is the only technique to distinguish between uni- and multi-lamellar vesicles [46]. Furthermore, facetted gel-phase vesicles and smooth liquid crystalline vesicles can easily be discriminated [47], whereas they are indistinguishable after drying. In the cryoTEM image, vesicles appear as circular objects with strong contrast at the rim. This can be explained by the fact that the projected thickness of the lipid bilayer shell is highest at the edges. However, the projection of a flat bilayer disc in face-on orientation leads to an equal contrast of the circular object. Thus, discs and vesicles can be easily differentiated. The smallest self-assembled soft-matter aggregates that can be visualized by cryoTEM are globular detergent micelles with a diameter of about 5 nm [48], which are not detectable in stained samples.

## 3. (Cryo)TEM of Self-Assembled Fluorinated Amphiphiles

Classical phospholipids are amphiphilic molecules with one hydrophilic headgroup and two lipophilic tails. By replacing one or several hydrogen atoms in the tail region with fluorine atoms, the self-assembly properties of these fluorinated lipids change considerably. Since the early 1980s, the synthesis and self-assembly of fluorinated lipids have been of continuous interest, especially because of the increased stability of the vesicles thus formed [49,50,51] that became suitable for drug-delivery applications [52]. The shape of the vesicles was visualized by negative stain, freeze–fracture, and cryoTEM [52,53,54,55].

**Figure 4:**
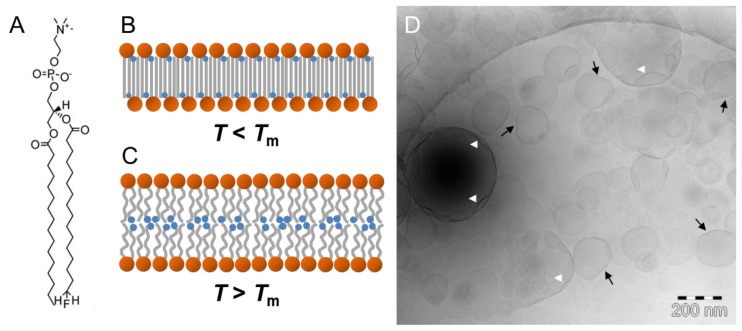
(**A**) Chemical structure of 1-palmitoyl-2-[16-fluoropalmitoyl]-*sn*-glycero-3-phosphocholine (F-DPPC). (**B**,**C**) Schematic model of F-DPPC lamellar aggregates in aqueous suspension forming an interdigitated L_β_ phase below *T*_m_ (**B**) and a liquid crystalline phase L_α_ above *T*_m_ (**C**). (**D**) CryoTEM image of an F-DPPC suspension after preparation above *T*_m_ and 24 h storage below *T*_m_. Black arrows point to planar vesicle regions formed due to F-DPPC interdigitation, and white arrow heads indicate invaginations due to an increased F-DPPC area during phase transition caused by interdigitation at constant water volume of the vesicle. Figure adapted from references [57,58].

Upon incorporation of a single fluorine atom at the end of the *sn*-2 chain of 1,2-dipalmitoyl-*sn*-glycero-3-phosphocholine (DPPC), no change of the lipid alkyl chain packing was expected. Instead, a rather classical bilayer arrangement with the fluorine atoms residing near the center of the lipid bilayer was anticipated. However, X-ray diffraction and solid-state NMR experiments clearly confirmed that 1-palmitoyl-2-[16-fluoropalmitoyl]-*sn*-glycero-3-phosphocholine (F-DPPC) (Figure 4A) forms vesicles with a lipid bilayer in the normal liquid–crystalline L_α_ phase above the transition temperature *T*_m_, but a fully interdigitated lipid layer in the gel phase L_β_ below *T*_m_ (Figure 4B,C) [56,57]. Mahrhauser et al. applied freeze–fracture TEM to visualize F-DPPC vesicles, but no information about the lipid layer thickness could be obtained [52]. Recently, Shah investigated the potential of synthetic phospholipids such as F-DPPC as membrane mimics during interaction with amphiphilic and polyphilic block copolymers [58]. He performed cryoTEM of vitrified 100 nm F-DPPC vesicles after extrusion above *T*_m_ of 50 °C and subsequent storage below *T*_m_ for at least 24 h. In Figure 4D, the cryoTEM image shows uni-lamellar F-DPPC vesicles with diameters ranging from 100 to 400 nm. Obviously, some of the vesicles fused during storage. It is known that this fusion might be a consequence of the formation of a partially interdigitated phase [58]. It has also been observed that, under certain constraints, F-DPPC undergoes a frustrated or incomplete phase transition upon cooling [59]. This frustration might be the reason for the presence of large vesicles being composed of a mixed lipid packing arrangement. The consequences are seen in the unusual vesicle shapes. Whereas some of the small vesicles are flattened on one side (Figure 4D, black arrows), most probably because of the stiffness of the interdigitated lipid phase, larger vesicles show invaginations (Figure 4D, white arrow heads). During the transformation of a lipid bilayer into the interdigitated phase, the lipid layer increases in area followed by a potential increase in vesicle diameter. However, the water volume within the original vesicle is no longer sufficient to fill the larger vesicle, so that invagination occurs.

Apart from fluorinated lipids, fluorinated surfactants are an important class of amphiphiles because of their potential pharmaceutical application for drug delivery, ultrasound imaging, and preparation of microemulsions, stable injectable emulsions, gels, contrast agents, and vaccines [60,61]. Fluorinated amphiphiles are composed of two or three parts of various affinities for water and oil: a polar head, which is hydrophilic and lipophobic, a fluorocarbon chain, which is hydrophobic and lipophobic, and, optionally, a hydrophobic and lipophilic hydrocarbon chain [62]. In water, these amphiphiles adopt a variety of supramolecular assemblies with diverse morphologies such as vesicles, fibers, or tubules, while their non-fluorinated analogs form micelles exclusively. Fluorinated surfactants are significantly more surface-active than their hydrocarbon analogues. In the past 20 years, alternative and mild surfactants have been developed; they were designed to keep membrane proteins soluble and active after solubilization [63,64,65,66]. On the one hand, these surfactants have fluorinated alkyl chains that are bulkier and more rigid than their hydrogenated counterparts, and on the other hand, fluorinated alkyl chains have little affinity for the hydrogenated surfaces of transmembrane protein segments, so they compete less efficiently with protein-protein interactions [67]. Until the late 1990s, fluorinated surfactants were shown not to be able to extract membrane proteins from biological membranes [60]. Later, it was found that fluorinated surfactants with different chemical structures were indeed capable of extracting membrane proteins, but only inefficiently. More recent experiments show that fluorinated surfactants may even stabilize membrane proteins by association with the outer transmembrane surface without disrupting the arrangement of the transmembrane helices by intercalation [67].

**Figure 5:**
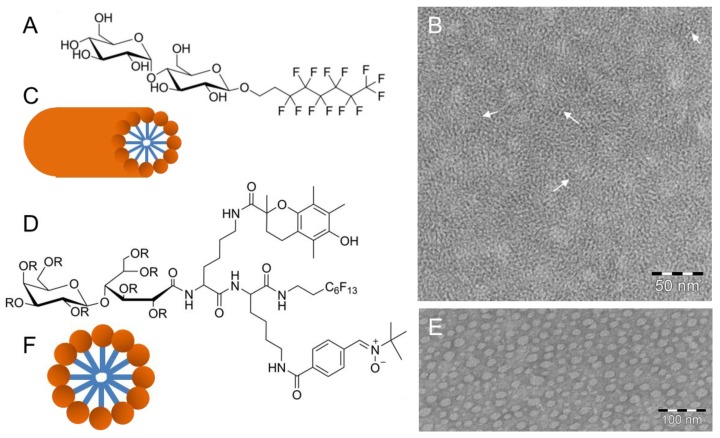
(**A**) Chemical structure of the nonionic fluorinated octyl maltoside derivative (F_6_OM). (**B**) TEM image of a negatively stained sample showing a dense fiber network. (**C**) Schematic model of a rod-shaped micelle. (**D**) Chemical structure of FATxPBN with R = H. (**E**) TEM image of a negatively stained sample showing large globular micelles. (**F**) Schematic model of a spherical micelle. Figure adapted from references [68,69]; PBN: *α*-phenyl-*N*-*tert*-butylnitrone; FATxPBN: PBN-Trolox conjugate (Trolox: 6-hydroxy-2,5,7,8-tetramethylchroman-2-carboxylic acid).

Recently, Frotscher et al. [68] demonstrated the suitability of a fluorinated surfactant for the isolation of membrane proteins. By using the nonionic fluorinated octyl maltoside derivative F_6_OM (Figure 5A), they successfully solubilized 1-palmitoyl-2-oleyl-*sn*-glycero-3-phosphocholine (POPC) vesicles and refolded the outer membrane phospholipase A (OmpLA) into POPC vesicles generating enzymatically active proteoliposomes. Above its critical micellar concentration, the surfactant F_6_OM alone self-assembles into elongated, up to 100 nm long, rod-shaped micelles observable by negative-stain TEM [68]. Figure 5B shows a dense network of intertwined flexible rods, with a diameter of about 2–3 nm, suggesting a molecular arrangement of F_6_OM within the rods described in Figure 5C. The ratio of the headgroup to chain volume would suggest a spherical micelle as the optimal aggregate shape. However, non-spherical cylindrical shapes of F_6_OM aggregates were observed. Obviously, the more rigid fluorinated chains prevent the formation of spherical micelles.

Amphiphilicity is also a key feature for bioactivity and protection against in vitro and in vivo oxidative toxicity, for example, light-induced retinal degeneration [70]. Rosselin et al. [69] synthesized the amphiphilic divalent antioxidant FATxPBN (Figure 5D) consisting of two lysine amino acids as a scaffold upon which two antioxidant moieties are grafted. An additional perfluorinated chain supplies additional hydrophobicity without inducing a cytolytic effect [71]. The polar headgroup is derived from the amine group of lysine. TEM investigations using negatively stained samples of aqueous solutions of FATxPBN above the critical micellar concentration showed the formation of spherical micelles (Figure 5E,F) with a diameter range between ~6 and ~25 nm. In the TEM images, the micelles appear mostly ellipsoidal, and the average diameter determined by computer-assisted imaging analysis is ~16 ± 2 nm, which is in agreement with dynamic light scattering data [69]. However, TEM clearly demonstrated a large variety in shape and size of the formed micelles.

## 4. (Cryo)TEM of Phospholipid Model Membranes Interacting with Amphiphilic Bolalipids, Amphiphilic T-Shaped Molecules, and X-Shaped Bolapolyphiles

### 4.1. Phospholipid Membranes and Amphiphilic Bolalipids

Bolalipids are amphiphilic molecules that are composed of two polar headgroups attached to one or two hydrophobic chains. Naturally occurring bolalipids stabilize archaebacterial membranes by spanning the lipid bilayer so that the bacteria withstand harsh environments such as high temperature and low pH-values [72]. Inspired by this concept, enormous efforts have been made to isolate and to synthesize bolalipids in order to apply them for vesicle stabilization in drug-delivery systems for pharmaceutical applications [24,73,74,75,76]. Since the isolation and synthesis of archaeal bolalipids are quite demanding, simplified artificial bolalipids with only one hydrophobic alkyl chain have been synthesized. Unfortunately, almost all of them failed to stabilize classical lipid bilayers because of packing problems within the hydrophobic membrane region [77].

**Figure 6:**
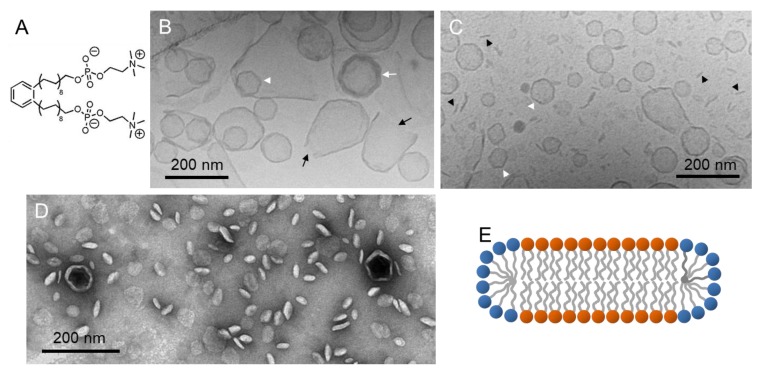
(**A**) Chemical structure of PC-C17oPhC17-PC. (**B**,**C**) CryoTEM images of a bolalipid-DPPC mixture with a molar ratio of bolalipid:DPPC = 1:10. White arrows point to facetted multi-lamellar vesicles, white arrowheads to uni-lamellar facetted vesicles, black arrows indicate open vesicles, and black arrowheads show discs observed edge-on. (**D**) TEM image of a negatively stained sample with the molar ratio of bolalipid:DPPC = 1:10. (**E**) Schematic model of mixed DPPC bilayer discs with bolalipids occupying the rim. Figure adapted from reference [78].

During their search for membrane-spanning bolalipids, Drescher et al. synthesized a single-chain bolalipid with phosphocholine headgroups and a *para*-substituted phenylene-modified alkyl chain PC-C17oPhC17-PC (Figure 6A) [78]. (Cryo)TEM experiments showed that a mixture of PC-C17oPhC17-PC with DPPC in a 1:10 molar ratio destabilizes DPPC vesicles (Figure 6B–D).

CryoTEM images of aqueous mixtures quenched from room temperature (Figure 6B,C) show the simultaneous presence of facetted multi-lamellar (white arrows) and uni-lamellar vesicles (white arrowheads). Facetted vesicles are observed when lipids are in the gel state due to the high membrane bending stiffness. Open vesicles (black arrows) as well as discs seen edge-on (black arrowheads) can also be found. The average disc size is 40 to 60 nm in diameter. Discs in different orientations (face-on, tilted, and edge-on) could also easily be visualized by TEM using negatively stained samples (Figure 6D), whereas closed and open vesicles could not be discriminated because of the drying procedure. A schematic model of the discs is depicted in Figure 6E showing a DPPC bilayer with bolalipid molecules accumulated at the rim of the bilayer discs stabilizing them against fusion. This example impressively demonstrates the ability of the TEM technique to simultaneously visualize various aggregate shapes with sizes between 40 and 400 nm.

### 4.2. Phospholipid Membranes and Amphiphilic T-Shaped Molecules

**Figure 7:**
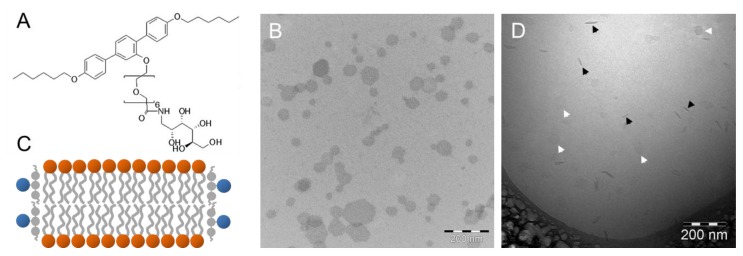
(**A**) Chemical structure of the T-shaped amphiphile A6/6. (**B**) TEM image of a negatively stained sample with a molar ratio of A6/6:DPPC = 1:10. (**C**) Schematic model of mixed DPPC bilayer discs with A6/6 occupying the rim. (**D**) CryoTEM image of the same A6/6-DPPC mixture. Black and white arrowheads indicate discs observed edge-on and face-on, respectively. Figure adapted from reference [81].

T-shaped facial amphiphiles are rod-like mesogens with alkyl chains at both termini and a polar group in the lateral position. Each of the building blocks, the rigid core, the terminal chains, and the lateral chain, have a tendency to segregate into their distinct own subspace [79]. The T-shaped amphiphilic molecule A6/6 (Figure 7A) forms a columnar hexagonal liquid–crystalline phase between the crystalline and the isotropic liquid when studied in bulk [80]. Because of the hydrophilic and flexible side chain attached to a rigid terphenyl core with terminal hexyloxy alkyl chains, it was expected that also that the formation of lyotropic phases could be possible.

Scholtysek et al. studied the interaction of A6/6 with DPPC membranes at a molar ratio of 1:10 [81]. They applied negatively stained samples and showed by TEM that the T-shaped facial amphiphile transforms DPPC vesicles into flat hexagonal sheets with dimensions of 50 to 200 nm. Figure 7B shows the discs in a uniform face-on orientation due to the drying procedure. The hexagonal shape is probably due to the lamellar gel phase of DPPC, where the tilted alkyl chains are packed in a hexagonal lattice. The sheets are, therefore, most probably rigid discs with A6/6 molecules located at the rim, where they shield the hydrophobic surfaces of the lipid bilayer (Figure 7C), but their exact orientation is still under discussion. The disc-like shape was verified by cryoTEM, where the discs are seen face-on (white arrowheads) or edge-on (black arrowheads). In the latter case, the discs appear as rod-like structures with a significantly higher electron density as compared to the discs viewed from the top (Figure 7D).

This example clearly shows that the disc shape of aggregates can be visualized unambiguously by cryoTEM, where the discs are trapped in different orientations. By contrast, negatively stained samples might preferentially visualize discs in the face-on orientation due to the drying. However, the increased contrast is an advantage during visualization, so that the hexagonal disc shape becomes discernable. This is not possible in cryoTEM, where the low electron density and a possible tilt of the lipid bilayer disc hamper an exact description of the disc shape.

### 4.3. Phospholipid Membranes and X-Shaped Bolapolyphiles

Polyphilic molecules are composed of at least three structural moieties that are characterized by three different affinities to water: (i) hydrophilic or lipophobic, (ii) hydrophobic or lipophilic, and (iii) mesogenic. Besides polyphilic polymers [82], X-shaped bolapolyphiles belong to this class of molecules exhibiting three different philicities [83]: a lipophilic, rigid, π–π stacking core; two flexible lipophilic side chains; and two hydrophilic, hydrogen-bonding head groups. Mixing experiments of X-shaped bolapolyphiles with DPPC giant uni-lamellar vesicles showed the formation of dendritic domains with hexagonal symmetry visualized by confocal microscopy [84].

**Figure 8:**
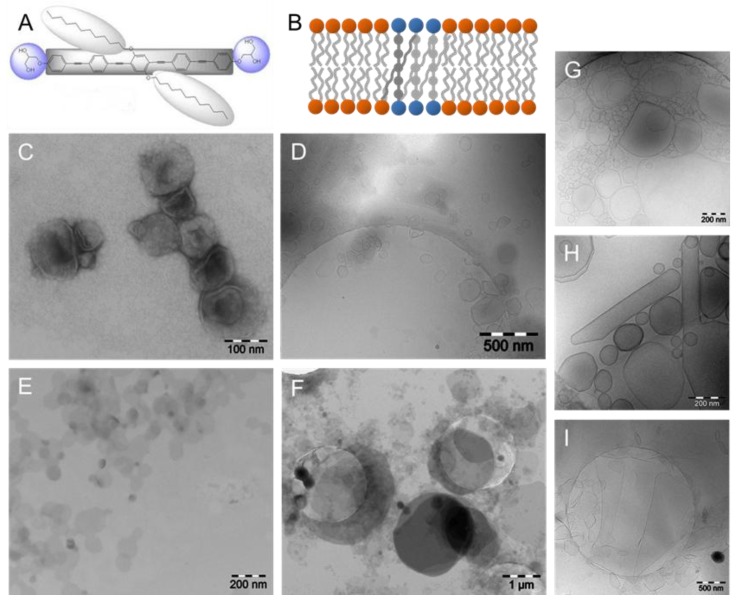
(**A**) Chemical structure of the X-shaped bolapolyphile B12. (**B**) Schematic model of phase-separated B12 in DPPC bilayers due to π–π interactions between B12 molecules. (**C**–**F**) TEM images of B12:DPPC = 1:10 (**C**,**D**), and B12:DPPC = 1:4 (**E**,**F**), respectively. TEM images of negatively stained samples are shown in (**C**,**E**), and cryoTEM images are given in (**D**,**F**). (**G**–**I**) CryoTEM images of B6:DPPC mixtures = 1:10 (**G**), 1:4 (**H**), and 1:1 (**I**) are shown for comparison. Figure adapted from references [36,85].

Lechner et al. investigated the miscibility of DPPC and B12, a bolapolyphile built of a rigid π-conjugated oligo (phenylene ethynylene) backbone with two laterally attached flexible and lipophilic alkyl chains (*n*-OC_12_H_25_) at opposite sides of the central benzene ring of the rodlike core and terminated by hydrophilic glycerol groups at both ends (Figure 8A) [85]. A negatively stained sample of a 1:10 mixture of B12 and DPPC showed collapsed vesicles (Figure 8C) resulting from drying during sample preparation. The corresponding cryoTEM sample quenched from 22 °C confirmed the presence of vesicles with diameters of 50 to several hundred nanometers (Figure 8D). Most vesicles are facetted as expected for gel-phase lipids. An increasing amount of B12 within the mixture with DPPC (1:4) resulted in completely different aggregate shapes. Both stained and vitrified samples showed the formation of flat discs with diameters up to several micrometers in diameter (Figure 8E,F). Whereas the EM image of the stained sample showed discs with diameters between 40 and 200 nm (Figure 8E), the vitrified sample gave larger round shaped and stiff sheets covering the 2 µm holes of the carbon film of the grid (Figure 8F). These differences in size might be due to the different preparation procedure of negatively stained and cryoTEM samples or to a different age of the samples, so that small discs could fuse with time. Whereas negatively stained specimens were made immediately after sample preparation of the B12/DPPC mixture, cryo preparation was performed one day afterwards. Figure 8B gives a schematic model of phase-separated B12 molecules in DPPC bilayers based on results from differential scanning calorimetry, IR spectroscopy, and X-ray diffraction experiments at room temperature. However, domain formation could not be verified by TEM because of the marginal difference in electron density between the domains.

The tendency to form discs seems to be strongly dependent on the length of the lipophilic alkyl chains (*n*-OC_n_H_n+1_) at the central benzene ring of the rod-like core. It is expected that shorter chains will not disturb the DPPC bilayer so efficiently. The bolapolyphile B6 has significantly shorter alkyl chains (*n*-OC_6_H_13_) than B12 and in mixtures with DPPC, vesicles are stable up to a molar ratio of B6:DPPC = 1:1. CryoTEM showed that with increasing amount of B6, the facetted vesicles become larger and transform into elongated straight tubes (Figure 8G,H). At a 1:1 molar ratio, vesicles coexist with extended and foldable sheets up to 2 µm in size (Figure 8I). So, even if TEM was not able to discriminate between DPPC and B12 domains, it delivers important information about the size, shape, and stiffness of vesicular aggregates in the nanometer to micrometer range not achievable by other techniques.

## 5. (Cryo)TEM of Phospholipid Model Membranes Interacting with Amphiphilic Macromolecules

Amphiphilic macromolecules, such as di- and tri-block copolymers, are well-studied classes of synthetic macromolecules binding to lipid membranes driven by polar interactions in the lipid headgroup region [86] and/or hydrophobic interactions through incorporation into the hydrophobic inner region of the membrane [87]. Recently, a novel class of polyphilic triblock copolymers has been synthesized [88,89,90] and a triphilic copolymer was investigated with respect to its interaction with lipid membranes [82]. Schwieger et al. showed that the perfluoro moieties of the perfluoroalkyl end-capped triblock copolymer F_9_-PGMA_20_-PPO_34_-PGMA_20_-F_9_ induce the tendency for phase separation [82]. It is known that the miscibility of hydrogenated and fluorinated compounds is non-ideal and unfavorable, since the dispersion forces between fluorinated chains are much weaker than those between hydrogenated chains. As a consequence, the incorporation of triblock-copolymers with hydrocarbon and fluorocarbon tails strongly depends on the interactions between the hydrophilic headgroups of the lipids and the hydrophilic polymer blocks [91,92].

**Figure 9:**
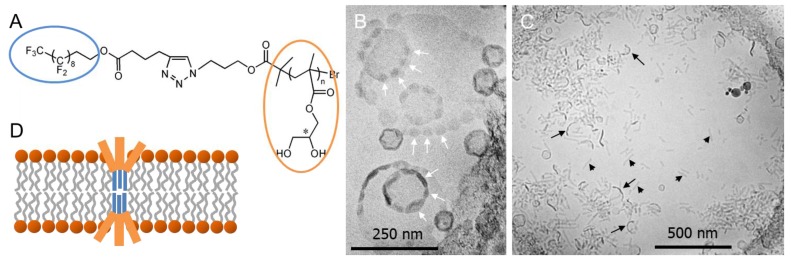
(**A**) Chemical structure of PGMA_14_-F_9_ with *n* = 14. (**B**,**C**) CryoTEM images of PGMA_14_-F_9_:DPPC = 1:10 mixtures. White arrows point to F-enriched phase-separated regions in vesicles; black arrows show open vesicle parts, and black arrowheads indicate disc or fiber aggregates. (**D**) Schematic model of phase-separated PGMA_14_-F_9_ in l-DPPC bilayers. Figure adapted from references [93,94].

Scholtysek et al. described the miscibility of PGMA_14_-F_9_ (Figure 9A) with l-DPPC bilayers using differential scanning calorimetry [93]. Their observations indicated that the polymer is only marginally incorporated into lipid bilayers in the liquid–crystalline phase. However, cryoTEM clearly demonstrated a pronounced effect of PGMA_14_-F_9_ on the bilayer integrity. Figure 9B shows intact vesicles with diameters between 50 and 400 nm. The rim of the vesicles exhibits an unequal distribution of the electron density indicated by dark spots (white arrows). This is most probably a result of the phase-separated perfluoroalkyl chains of PGMA_14_-F_9_ due to the significantly higher electron density of the fluorine atoms compared to that of hydrogen. The interaction of this polymer with l-DPPC bilayers is so strong that most of the vesicles break apart into smaller aggregates (Figure 9C). The shape of the formed aggregates could not be determined unambiguously, most probably these are vesicle fragments (black arrows) and eventually fibers or discs (black arrowheads). In Figure 9D, a schematic model of phase-separated PGMA_14_-F_9_ in l-DPPC is shown, where the stiff perfluoroalkyl chains insert into the lipid bilayer. However, they are too short to span one membrane leaflet, inducing packing problems within the bilayer that destabilize the membrane.

**Figure 10:**
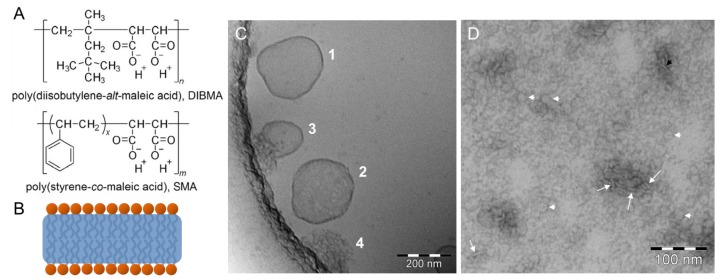
(**A**) Chemical structures of alternating diisobutylene/maleic acid (DIBMA) and styrene/maleic acid (SMA). (**B**) Schematic model of a copolymer/lipid particle. (**C**) CryoTEM image showing the process of solubilization of 1-palmitoyl-2-oleyl-*sn*-glycero-3-phosphocholine (POPC) vesicles during incubation with SMA, indicating intact deformed vesicles (1), deformed and perforated vesicles (2), crushing vesicles (3), and crushed vesicles (4). (**D**) TEM image of a negatively stained sample with a molar ratio of DIBMA:DMPC = 1:10, white arrows and arrowheads indicate discs observed edge-on and face-on, respectively. Figure adapted from reference [101].

Another class of amphiphilic polymers are styrene/maleic acid (SMA) copolymers which solubilize membrane proteins and surrounding lipids to form SMA/lipid particles (SMALPs) [95,96,97] (Figure 10A). These SMALPs are disc-shaped nanoparticles with diameters of 10–25 nm made up of a lipid-bilayer patch bounded by a polymer belt [98] (Figure 10B). During the solubilization process, SMA mediates the disruption of lipid vesicles by penetration of its hydrophobic styrene moieties into the hydrocarbon core of the membrane. It seems that the formation of the bilayer patches sets in with very low SMA content in the membrane [99,100]. Scheidelaar et al. showed the formation of intermediate vesicular structures and open bilayer fragments using cryoTEM [99]. The cryoTEM image in Figure 10C indicates different intermediate aggregates in the coexistence range of POPC vesicle solubilization by SMA (S. Keller and A. Meister, unpublished results): an intact but deformed vesicle (1), a deformed and perforated vesicle (2), a collapsing vesicle together with SMALPs (3), and SMALPs after complete vesicle transformation (4). However, the contrast of vitrified samples of the suspended nanodiscs is very low, so that negatively stained samples might be advantageous. This technique has been applied by Oluwole et al. for the visualization of nanodiscs made by solubilization of 1,2-dimyristoyl-*sn*-glycero-3-phosphocholine (DMPC) vesicles with an alternating diisobutylene/maleic acid (DIBMA) copolymer (Figure 10A) [101]. The formed DIBMA/lipid particles (DIBMALPs) are seen in Figure 10D face-on as round-shaped discs (white arrowheads) and edge-on as stacks of discs (white arrows). In contrast to SMALPs, these DIBMALPs show only weak UV absorption bands. In addition, they are compatible with elevated concentrations of Mg^2+^ or Ca^2+^, thus allowing a quantification of incorporated membrane proteins and protein assays requiring divalent cations, respectively.

Recently, a novel type of amine-modified SMA polymer has been introduced that is also capable of encapsulating membrane proteins in nanodiscs [102]. Using negative staining, the formation of nanodiscs with diameters between 10 and 50 nm was reported depending on the lipid to SMA ratio. Whereas the small nanodiscs can be used for solution NMR spectroscopy studies, larger nanodiscs can be magnetically aligned for solid-state NMR studies on membrane proteins.

## 6. Conclusions

(Cryo)TEM of negatively stained or vitreous samples have emerged as useful methods for characterizing model membranes. Changes in lipid membrane shape and size and, occasionally, even phase separation within the membrane system can be clearly shown. This also includes changes brought about by the addition of amphiphilic or polyphilic molecules interacting with lipid membranes in various manners: attachment to or insertion into the lipid headgroup region due to hydrophilic interactions, or insertion into one or two membrane leaflets due to hydrophobic interactions with the inner membrane part. Whereas larger structures, such as vesicles, are easily visualized by cryoTEM, smaller aggregates, such as nanodiscs, have a low contrast in cryoTEM, so the staining process is an advantageous complementary method. It is also possible to trap intermediate states in vesicle-to-nanodisc transitions, where different aggregate shapes and sizes are visible, thus providing information usually not available from scattering techniques.
